# Assessment of *RET/PTC1 *and *RET/PTC3 *rearrangements in fine-needle aspiration biopsy specimens collected from patients with Hashimoto's thyroiditis

**DOI:** 10.1186/1756-6614-4-5

**Published:** 2011-01-10

**Authors:** Anna Cyniak-Magierska, Katarzyna Wojciechowska-Durczyńska, Kinga Krawczyk-Rusiecka, Arkadiusz Zygmunt, Andrzej Lewiński

**Affiliations:** 1 Department of Endocrinology and Metabolic Diseases, Medical University of Lodz, Poland; 2 Polish Mother's Memorial Hospital - Research Institute, Lodz, Poland

## Abstract

**Background:**

*RET/PTC *rearrangements are the most frequent molecular changes in papillary thyroid carcinoma (PTC). So far, 15 main *RET/PTC *rearrangements have been described, among which *RET/PTC1 *and *RET/PTC3 *are the most common in PTC - especially in radiation-induced tumours. *RET/PTC1 *and *RET/PTC3 *are the result of intrachromosomal paracentric inversions in chromosome 10, where *RET *and the activating genes (*H4 *and *ELE1*, respectively) are located. Recently, *RET/PTC *rearrangements have been shown not only in PTC but also in benign thyroid lesions, including Hashimoto's thyroiditis (HT). The aim of study was an assessment of *RET/PTC1 *and *RET/PTC3 *rearrangements in patients with Hashimoto's thyroiditis.

**Materials and methods:**

Thyroid aspirates, eligible for the study, were obtained from 26 patients with Hashimoto's thyroiditis by fine-needle aspiration biopsy (FNAB). Each aspirate was smeared for conventional cytology, while its remaining part was immediately washed out of the needle. The cells, obtained from the needle, were used in further investigation. Total RNA from FNAB was extracted by use of an RNeasy Micro Kit, based on modified Chomczynski and Sacchi's method and reverse transcription (RT-PCR) was done. Quantitative evaluation of *RET/PTC1 *and *RET/PTC3 *rearrangements by real-time PCR was performed by an ABI PRISM^® ^7500 Sequence Detection System. In the study, PTC tissues with known *RET/PTC1 *and *RET/PTC3 *rearrangements served as a reference standard (calibrator), while *β-actin *gene was used as endogenous control.

**Results:**

Amplification reactions were done in triplicate for each examined sample. No *RET/PTC1 *and *RET/PTC3 *rearrangements were found in the examined samples.

**Conclusions:**

Our results indicate that *RET/PTC1 *and *RET/PTC3 *rearrangements in Hashimoto's thyroiditis, if any, are rather rare events and further investigations should be conducted in order to determine molecular changes, connecting Hashimoto's thyroiditis with PTC.

## Background

Chromosomal rearrangements involving *RET *receptor tyrosine kinase (TK) protooncogene (*RET/PTC*) are a specific feature of papillary thyroid carcinoma (PTC). The *RET *protooncogene is activated by fusion of 3'-terminal portion of *RET*, coding for TK domain, with the 5' terminal region of different unrelated genes, leading to constitutive activation of the *RET *TK [1]. There are - at least -15 different *RET/PTC *rearrangements [2], but the two: *RET/PTC1 *and *RET/PTC3 *are the most common ones [3]. *RET/PTC1 *is formed by fusion with the *H4 *gene [1], and *RET/PTC3 *by fusion with the *ELE1 *(also designed *NCOA4*, *RFG *or *ARA70*) gene [4]. However, apart from *RET/PTC1 *and *RET/PTC3*, all the other variants of rearrangements do not seem to have an important role in the pathogenesis of PTC since they have been isolated just in rare cases [5].

Chromosomal rearrangements of *RET *in PTC were found with different frequency, depending on geographic variability and studied population. The reported frequency of *RET/PTC *in PTC varies from 0 to 87% [6]. It is interesting that the frequency of *RET/PTC *rearrangements not only depends on geographical region but also on applied technique of identification [6].

The relatively high prevalence of *RET/PTC *rearrangements was described in tumours associated with radiation exposure [7]. The higher prevalence of *RET/PTC3 *activation in PTC was observed in the contaminated areas after Chernobyl explosion [7]. The analysis of PTC, which developed after a long latency period after explosion showed higher prevalence of *RET/PTC1 *rearrangements [8]. This can suggest that *RET/PTC3 *rearrangements may be typical for radiation-induced childhood PTC with a short latency period, while *RET/PTC1 *rearrangements may be a marker for later-occurring PTC of radiation-exposed children and adults [8]. *RET/PTC *rearrangements have not been found in follicular thyroid carcinomas and anaplastic thyroid carcinomas [9].

Recently, *RET/PTC *rearrangements have been shown not only in PTC but also in Hürthle thyroid adenomas and carcinomas and also in Hashimoto's thyroiditis (HT) [10,11].

The prevalence of *RET/PTC *rearrangements in HT is still controversial. The study aimed at estimating the frequency of rearrangements of *RET *protooncogene in HT in the Polish population.

## Materials and methods

The Ethical Committee of the Medical University of Lodz approved the studies in this paper.

### Patients

Thyroid aspirates, eligible for this study, were obtained from patients undergoing FNAB for Hashimoto's thyroiditis. All tissue samples were taken after patients' informed consent was obtained. FNAB specimens from 26 patients (25 women, 1 man) were examined. Data characterising the patients involved in the study are presented in Table 1. The mean age of patients was 52.8 ± 15.4 years (mean ± SD), ranging from 18 to 88 years.

**Table 1:** Characteristics of HT patients involved in the study.

Case number	Sex	Age	Diagnosis
1	F	68	HT

2	F	67	HT

3	F	48	HT

4	F	45	HT

5	F	37	HT

6	F	68	HT

7	F	18	HT

8	F	53	HT

9	F	31	HT

10	F	52	HT

11	F	63	HT

12	F	54	HT

13	F	83	HT

14	F	88	HT

15	F	39	HT

16	F	50	HT

17	F	51	HT

18	F	41	HT

19	F	41	HT

20	F	49	HT
21	F	38	HT

22	F	65	HT

23	F	51	HT

24	F	54	HT

25	F	63	HT

26	M	57	HT

Thyroiditis was diagnosed, according to clinical symptoms and signs, together with data of hormonal and immunological tests and the results of cytological evaluation of FNAB specimens, collected under ultrasound guidance.

Ultrasound-guided FNAB was performed with the use of a 22-gauge needle and each aspirate was smeared for conventional cytology, while the remaining material was immediately washed out of the needle with 350 μl of RLT lysis buffer, containing β-mercaptoethanol and guanidine thiocyanate, used for RNA isolation (Qiagen, Germany). The samples were immediately frozen at -70°C and, thus, stored until total RNA isolation. The samples, containing visible blood contamination, were disqualified from further investigations.

### RNA extraction from FNAB

Total RNA from FNAB was extracted with the use of RNeasy Micro Kit (Qiagen, Germany). The manufacturer's instructions were followed, except that the cell lysate was applied for the second time to the column before the wash-step was carried out. The quality and quantity of total RNA was spectrophotometrically measured and - for some samples - with an Agilent 2100 Bioanalyzer.

### Reverse transcription

Reverse transcription (RT) was performed, using the whole RNA, extracted with TaqMan^® ^Reverse Transcripton Reagents (Applied Biosystems, Branchburg, New Jersey, USA). RT reaction mixture contained 5 μl of 10 × TaqMan RT Buffer (100 mM Tris-HCl pH 8.3, 500 mM KCl), 11.0 μl 25 mM MgCl_2_, 1.25 μl Multi Scribe™ reverse transcriptase (50 U/μl), 2.5 μl 50 μM oligo(dT)_16_, 1.0 μl RNase Inhibitor - RI (20 U/μl), and 10 μl 10 mM dNTPs in a total volume of 50 μl. RNAs were incubated for 10 minutes at 25°C, followed by 30 minutes at 48°C and 5 minutes at 95°C to inactivate reverse transcriptase (Multi Scribe™). Negative controls were performed simultaneously to eliminate a possible genomic DNA contamination (no reverse transcriptase reactions), as well as to eliminate a possible contamination of RT reagents (no RNA-added reactions).

### Real-time quantitative PCR

An established Relative Quantification Polymerase Chain Reaction (RQ-PCR) assay for *RET/PTC1 *and *RET/PTC3 *mRNA expressions, using the relative quantification method (ΔΔC_T_) were conducted in ABI PRISM 7500 Sequence Detection System (Applied Biosystems), according to the manufacturer's protocol. The PCR reactions for *RET/PTC1 *and *RET/PTC3 *rearrangements of genes were run with 50 ng of cDNA in a total volume of 50 μl, using TaqMan^® ^Universal PCR Master Mix (Applied Biosystems, Foster City, CA, USA) and the predesigned and labelled primer/probe set (Applied Biosystems, Foster City, CA, USA). Sequences of primers and probes used for this study were reported and validated according to Rhoden's *et al*. protocol (Table 2) [12]. After initial incubation at 50°C for 2 min to allow uracil-N-glycosylase (UNG) digestion, and at 95°C for 10 min to activate the AmpliTaq Gold^®^DNA polymerase, both of them were provided by the Universal PCR Master Mix, the samples were amplified through 45 biphasic cycles of 95°C for 15 sec and 60°C for 1 min (Table 3).

**Table 2:** The sequences of probes and primers of *RET/PTC1 *and *RET/PTC3 *gene rearrangements [12].

Gene	Amplicon (bp)	Forward primer	Reverse primer	Probe sequence
*RET/PTC1*	66	CGCGACCTGCGCAAA	CAAGTTCTTCCGAGGGAATTCC	*6FAM*-CAAGCGTAACCATCGAGGATCCAAAGT-*TAMRA*

*RET/PTC3*	81	CCCCAGGACTGGCTTACCC	CAAGTTCTTCCGAGGGAATTCC	*6FAM-*AAAGCAGACCTTGGAGAACAGTCAGGAGG-*TAMRA*

**Table 3:** Amplification conditions for *RET/PTC1 *and *RET/PTC3 *rearrangements using real-time PCR.

Times and temperatures			
		**Each of 45 cycles**	
		
**Initial setup**		**Denaturation**	**Annealing/Elongation**

HOLD	HOLD	CYCLE	

UNG activation 2 min, 50°C	10 min, 95°C	15 s, 95°C	1 min, 60°C

In the study, PTC tissues with known *RET/PTC1 *and *RET/PTC3 *rearrangements served as a reference standard (calibrator) [13]. Amplification reactions were done in triplicate for each sample (cDNA from the same PCR reaction but in separate wells). Controls with no template cDNA were used with each assay (negative control).

The expression levels of *β-actin *gene (*ACTB*) were measured, as endogenous control (reference gene), using the appropriate Assays-on-Demand™ Gene Expression product (Hs99999903_ml, Applied Biosystems Foster City, CA, USA).

Both gene expressions were measured for each thyroid lesion sample in the same PCR reaction but in separate wells.

**Figure 1:**
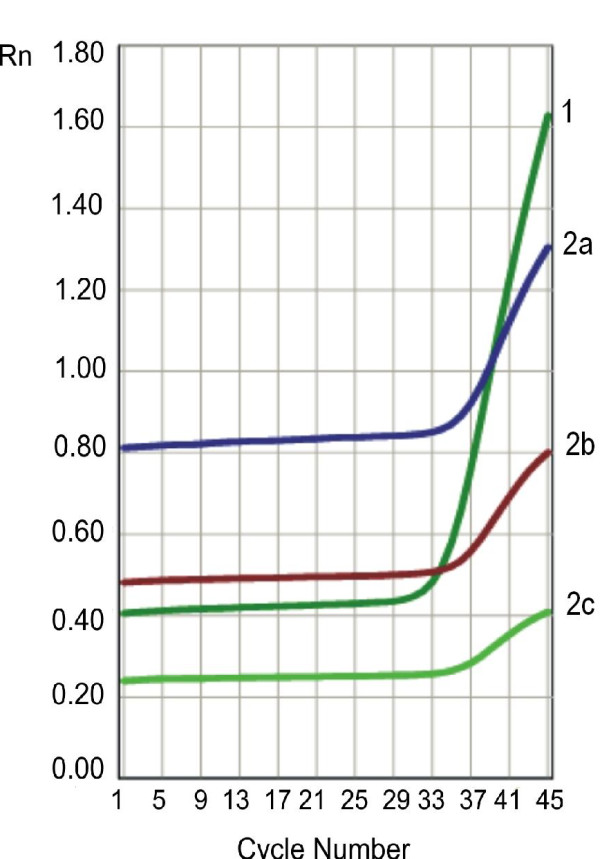
**Representative amplification curves for *β-actin *gene (curve no. 1) and *RET/PTC1 *rearrangement (curves nos. 2a, 2b, 2c) in PTC specimen**.

**Figure 2:**
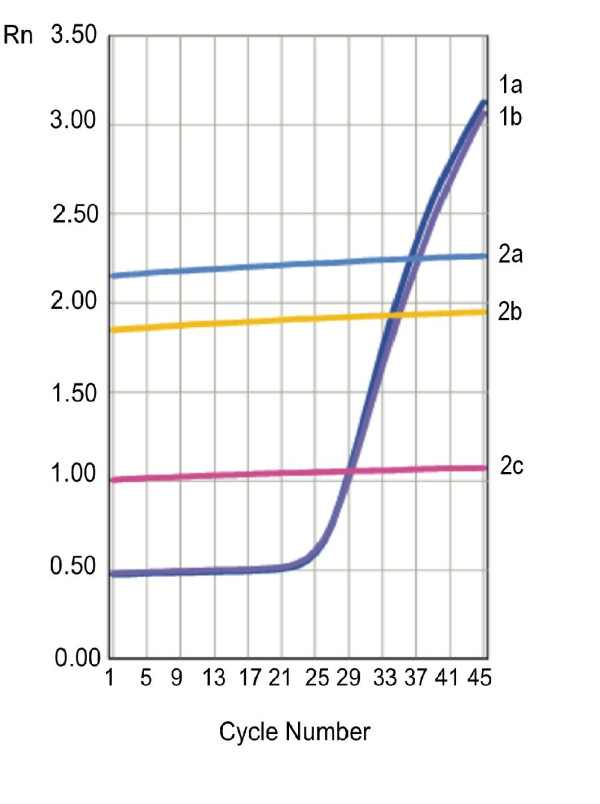
**Representative amplification curves for *β-actin *gene (curves nos. 1a, 1b) and *RET/PTC1 *rearrangement (curves nos. 2a, 2b, 2c) in HT specimen**.

Results were presented as Rn (normalized receptor) versus cycle graph, where Rn is the fluorescence of the receptor dye, divided by fluorescence of a passive reference dye (Figure. 1 and 2).

## Results

The specimens were amplified in the ABI PRISM 7500 Sequence Detection System in reaction, containing primers and probes for *RET/PTC1 *and *RET/PTC3 *rearrangements and a control gene, *β-actin*. The Sequence Detection System software, provided with the instrument, analyses the fluorescence data, generated during the reaction, and calculates the cycle number at which fluorescence crosses the threshold value (C_T_). Amplification reactions were done in triplicate for each examined sample.

No *RET/PTC1 *and *RET/PTC3 *rearrangements were found in the examined HT specimens (for *RET/PTC1 *- see Figure. 1 and 2; for *RET/PTC3 *- data not shown).

## Discussion

The prevalence of *RET/PTC *rearrangements in Hashimoto's thyroiditis remains a subject of controversy. Some authors have described activation of *RET *protooncogene in the majority number of HT patients. Sheils *et al*. [14] have shown the presence of *RET/PTC1 *rearrangement in a significant number of HT cases (95%). The majority of specimens have shown no evidence of concomitant PTC [14]. Wirtschafter *et al*. also have documented the presence of transcripts of *RET/PTC1 *and *RET/PTC3 *in 95% cases of HT [15]. Their study has suggested that multiple, independent occult PTCs exist in patients with HT at high frequency and *RET/PTC1 *and *RET/PTC3 *are early molecular markers of microcarcinoma. However, both of these studies had technical limitations, the authors used highly degraded RNA which was isolated from formalin-fixed and paraffin-embedded tissues. Kang *et al*. [16] have investigated the expression of RET, RAS and ERK proteins in oxyphilic cells in HT. They have shown greatly enhanced immunoexpression of these proteins, suggesting a molecular link between oxyphilic cell metaplasia in HT and the progression of PTC [16].

Rhoden *et al*. [11] have analyzed *RET/PTC *oncogenic activation in HT, PTC, oncocytic tumours and in normal thyroid samples using three independent techniques: real-time RT-PCR, laser capture microdissection (LCM) RT-PCR, and fluorescence *in situ *hybridization (FISH) method. In their study, normal samples have not shown *RET *protooncogene rearrangement. Sixty eight percent of HT were positive by FISH analysis, whereas only 17% HT have shown *RET/PTC *activation by real-time PCR. It is interesting that these conflicting results depended only on the method used in the study [11]. Despite some technical controversies (RT-PCR should be typically more sensitive method of detection than FISH), the importance of this paper is expressed through providing additional evidence for the possibility of presence *RET/PTC *rearrangements in a subpopulation of cells within the thyroid gland affected by HT.

On the other hand, other groups have not detected *RET *rearrangements in histologically benign thyroid tissue with HT. Moreover, they have not shown *RET/PTC *activation in PTCs arising on the background of HT, in contrast to PTC not associated with that disease [17]. Also, Sadow *et al*. have not identified *RET/PTC1 *and *RET/PTC3 *in dominant nodules from Hashimoto's goitre, supporting the notion that these nodules are neither malignant nor precursor lesions of PTC [18].

In our study, we have performed real-time relative PCR assay for *RET/PTC1 *and *RET/PTC3 *rearrangements, based on fluorescent TaqMan methodology and using the ABI PRISM 7500 Sequence Detection System (Applied Biosystems). Real-time PCR is a sensitive, precise technique for detection of nucleic acids. Unfortunately, we have not found *RET/PTC1 *and *RET/PTC3 *rearrangements in the examined samples of patients with HT. On one hand, it could be a result of the limited number of cases of HT in our study, on the other, it could suggest that the association between Hashimoto's thyroiditis and follicular cell-derived thyroid tumours, as well as *RET/PTC *rearrangements as a putative link between them, remain still controversial.

In summary, the results of our study indicate that *RET/PTC1 *and *RET/PTC3 *rearrangements in Hashimoto's thyroiditis, if any, are rather rare events and further investigations seem to be necessary to determine molecular changes associating Hashimoto's thyroiditis with PTC.

## Competing interests

The authors declare that they have no competing interests.

## Authors' contributions

AC-M designed and coordinated the study, carried out the molecular genetic studies and drafted the manuscript. KW-D and KR-K participated in performing molecular studies. AZ participated in coordination of the study. AL, the senior author, wrote the manuscript. All authors have read and approved the final manuscript.

## References

[B1] Grieco M Santoro M Berlingieri MT Melillo RM Donghi R Bongarzone I Pierotti MA Della Porta G Fusco A Vecchio G PTC is a novel rearranged form of the ret proto-oncogene and is frequently detected in vivo in human thyroid papillary carcinomas Cell 1990 60 557 563 10.1016/0092-8674(90)90659-3 2406025

[B2] Tallini G Asa SL RET oncogene activation in papillary thyroid carcinoma Adv Anat Pathol 2001 8 345 354 10.1097/00125480-200111000-00005 11707626

[B3] Nikiforov YE RET/PTC rearrangement in thyroid tumors Endocr Pathol 2002 13 3 16 10.1385/EP:13:1:03 12114746

[B4] Santoro M Dathan NA Berlingieri MT Bongarzone I Paulin C Grieco M Pierotti MA Vecchio G Fusco A Molecular characterization of RET/PTC3; a novel rearranged version of the RET proto-oncogene in a human thyroid papillary carcinoma Oncogene 1994 9 509 516 8290261

[B5] Fusco A Santoro M 20 years of RET/PTC in thyroid cancer: clinicopathological correlations Arq Bras Endocrinol Metabol 2007 51 731 735 17891236 10.1590/s0004-27302007000500010

[B6] Zhu Z Ciampi R Nikiforova MN Gandhi M Nikiforov YE Prevalence of RET/PTC rearrangements in thyroid papillary carcinomas: effects of the detection methods and genetic heterogeneity J Clin Endocrinol Metab 2006 91 3603 3610 10.1210/jc.2006-1006 16772343

[B7] Bounancer A Wicker R Caillou B Cailleux AF Sarasin A Schlumberger M Suarez HG High prevalence of activating *RET *proto-oncogene rearrangements in thyroid tumors from patients who had received external radiation Oncogene 1997 15 1263 1273 10.1038/sj.onc.1200206 9315093

[B8] Smida J Salassidis K Hieber L Zitzelsberger H Kellerer AM Demidchik EP Negele T Spelsberg F Lengfelder E Werner M Bauchinger M Distinct frequency of ret rearrangements in papillary thyroid carcinomas of children and adults from Belarus Int J Cancer 1999 80 32 38 10.1002/(SICI)1097-0215(19990105)80:1<32::AID-IJC7>3.0.CO;2-L 9935226

[B9] Tallini G Santoro M Helie M Carlomagno F Salvatore G Chiappetta G Carcangiu ML Fusco A RET/PTC oncogene activation defines a subset of papillary thyroid carcinomas lacking evidence of progression to poorly differentiated or undifferentiated tumor phenotypes Clin Cancer Res 1998 4 287 294 9516913

[B10] Chiappetta G Toti P Cetta F Giuliano A Pentimalli F Amendola I Lazzi S Monaco M Mazzuchelli L Tosi P Santoro M Fusco A The RET/PTC oncogene is frequently activated in oncocytic thyroid tumors (Hürthle cell adenomas and carcinomas), but not in oncocytic hyperplastic lesions J Clin Endocrinol Metab 2002 87 364 369 10.1210/jc.87.1.364 11788677

[B11] Rhoden KJ Unger K Salvatore G Yilmaz Y Vovk V Chiappetta G Qumsiyeh MB Rothstein JL Fusco A Santoro M Zitzelsberger H Tallini G RET/papillary thyroid cancer rearrangement in nonneoplastic thyrocytes: follicular cells of Hashimoto's thyroiditis share low-level recombination events with a subset of papillary carcinoma J Clin Endocrinol Metab 2006 91 2414 2423 10.1210/jc.2006-0240 16595592

[B12] Rhoden KJ Johnson C Brandao G Howe JG Smith BR Tallini G Real-time quantitative RT-PCR identifies distinct c-RET, RET/PTC1 and RET/PTC3 expression patterns in papillary thyroid carcinoma Lab Invest 2004 84 1557 1570 10.1038/labinvest.3700198 15502856

[B13] Brzeziańska E Karbownik M Migdalska-Sęk M Pastuszak-Lewandoska D Włoch J Lewiński A Molecular analysis of the RET and NTRK1 gene rearrangements in papillary thyroid carcinoma in the Polish population Mutat Res 2006 599 26 35 16483615 10.1016/j.mrfmmm.2005.12.013

[B14] Sheils OM O'Leary JJ Uhlmann V Lüttich K Sweeney EC ret/PTC-1 activation in Hashimoto thyroiditis J Surg Pathol 2000 8 185 189 10.1177/106689690000800305 11493988

[B15] Wirtschafter A Schmidt R Rosen D Kundu N Santoro M Fusco A Multhaupt H Atkins J Rosen MR Keane W Rothstein JL Expression of the *RET/PTC *fusion gene as a marker for papillary carcinoma in Hashimoto's thyroiditis Laryngoscope 1997 107 95 100 10.1097/00005537-199701000-00019 9001272

[B16] Kang DY Kim KH Kim JM Kim SH Kim JY Baik HW Kim YS High prevalence of RET, RAS, and ERK expression in Hashimoto's thyroiditis and in papillary thyroid carcinoma in the Korean population Thyroid 2007 11 1 7 10.1089/thy.2007.0035 17900235

[B17] Nikiforova MN Caudill CM Biddinger P Nikiforov YE Prevalence of *RET/PTC *rearrangements in Hashimoto's thyroiditis and papillary thyroid carcinomas Int J Surg Pathol 2002 10 15 22 10.1177/106689690201000104 11927965

[B18] Sadow PM Heinrich MC Corless CL Fletcher JA Nosé V Absence of BRAF, NRAS, KRAS, HRAS mutations, and RET/PTC gene rearrangements distinguishes dominant nodules in Hashimoto thyroiditis from papillary thyroid carcinomas Endocr Pathol 2010 21 73 79 10.1007/s12022-009-9101-3 20012784

